# Trait profile of maize varieties preferred by farmers and value chain actors in northern Ghana

**DOI:** 10.1007/s13593-021-00708-w

**Published:** 2021-07-06

**Authors:** Gloria Boakyewaa Adu, Baffour Badu-Apraku, Richard Akromah, Isaac Kodzo Amegbor, Desmond Sunday Adogoba, Alidu Haruna, Kulai Amadu Manigben, Paulina Abanpoka Aboyadana, Alexander Nimo Wiredu

**Affiliations:** 1 CSIR-Savanna Agricultural Research Institute, Nyankpala, Ghana; 2 International Institute of Tropical Agriculture, Ibadan, Nigeria; 3 College of Agriculture and Renewable Natural Resources, Kwame Nkrumah University of Science and Technology, Kumasi, Ghana; 4 Department of Plant Breeding, University of the Free State, P.O. Box 339, Bloemfontein, South Africa; 5 International Institute of Tropical Agriculture, Nampula, Mozambique

**Keywords:** Trait preference, Cultivar development, Variety release, Variety adoption, Grain yield

## Abstract

Plant breeders’ knowledge of precise traits preferred by variety users would accelerate varietal turnover and widen adoption of newly developed maize varieties in Ghana. The objective of this research was to provide empirical evidence of trait preferences of farmers and other actors in the maize value chain in northern Ghana, based on which research strategies for maize improvement could be formulated. Participatory rural appraisal was conducted in 2016 to determine key traits preferred by maize value chain actors across the three regions in northern Ghana. A total of 279 maize value chain actors were interviewed. Different scoring and ranking techniques were used to assess the maize traits preferred by the different actors. Participatory variety selection trials were also conducted in the Tolon, West Gonja, Binduri, and Sissala East districts in northern Ghana from 2014 to 2016. The mother-baby trial approach was used to evaluate eight hybrids with 3000 farmers. Data on yield and agronomic performance of the hybrids and farmer’s selection criteria were collected. Data analyses were performed using GenStat Edition 16 and SPSS Edition 20 statistical packages. The participatory rural appraisal method identified farmers, input dealers, traders, and processors as the primary maize value chain actors in the study areas. Trait preferences of the different actors overlapped and revolved around grain quality including nutritional value, and stress tolerance and grain yield. Results of the participatory variety selection study revealed that across districts, farmers preferred high-yielding varieties with multiple cobs per plant, white grain endosperm color, and bigger and fully filled cobs. For the first time, our holistic assessment of the trait preferences of key actors of the maize value chain in northern Ghana revealed a comprehensive list of traits, which could be used by breeders to develop varieties that may be preferred by all value chain actors in northern Ghana.

**Supplementary Information:**

The online version contains supplementary material available at 10.1007/s13593-021-00708-w.

## Introduction

Maize is one of the most important food crops in the world. Maize, rice, and wheat provide at least 30% of the food calorie needs of more than 4.5 billion people in 94 developing countries of the world (Shiferaw et al. 2011). In sub-Saharan Africa (SSA), “maize is life” due to its importance to food security and the economic well-being of maize consumers (Fisher et al. 2015). In Ghana, maize is an important food crop in the domestic market accounting for more than 50% of the country’s total cereal production (Ragasa et al. 2014). Maize is grown in all the regions of Ghana (WABS 2008). It is estimated that 85% of the total maize grown in Ghana is used for human consumption with the remaining 15% used for animal feed, mainly in the livestock and poultry industries (Angelucci 2012; Andam et al. 2012). According to the Millennium Development Authority (MiDA 2010), maize is the most important commodity crop in the country, second only to cocoa. Despite the importance of maize to Ghana’s economy, maize grain yield has been about 1.5 t ha^−1^ since 2000. This yield is far below the international output of maize (FAO 2016). The low maize output is due to the fact that maize production in Ghana is severely constrained by poor soil fertility combined with limited use of chemical fertilizers, erratic rainfall patterns, drought, and lack of access to improved seed and poor agronomic practices (WABS 2008; Etwire et al. 2013). The Ghanaian maize industry is also faced with low adoption of improved varieties and a low variety replacement rate. The average age of maize varieties currently on the market is 12.5 years (Mabaya et al. 2017). An open-pollinated variety, Obatanpa (released in 1992), is the oldest variety in the maize market. Obatanpa is popular because farmers like its attributes and also because other newer maize varieties have not been promoted as much as Obatanpa (Ragasa et al. 2013; Mabaya et al. 2017). Although new varieties are released almost every year by the National Research Institutes in collaboration with their International Research Partners (MoFA – SRID 2012), these improved maize varieties are poorly adopted partly due to the failure of the improved varieties to address farmers’ preferences and production constraints (Ragasa et al. 2013). Plant breeders have often been criticized for failing to consider the preferences of the consumers and the needs of the farmers especially those in marginal areas during the cultivar development process (Sibiya et al. 2013).

To accelerate the adoption of newly developed varieties, breeders should take into consideration the concerns and preferences of producers (farmers), consumers, and other actors at the early stage of cultivar development (De Groote et al. 2002). Participatory plant breeding approach involves bridging the communication gap between all stakeholders involved in the maize value chain. This allows plant breeders to interact with farmers and other actors to set breeding objectives and share responsibility for decision-making, implementation, and generation of products (Morris and Bellon 2004). Participatory plant breeding (PPB) approaches have been extensively used in cultivar development programs to address farmers’ needs and their socioeconomic situation as well as consumers’ preferences (Kamara et al. 2006; Tetteh et al. 2011; Dao et al. 2015; Ajambo et al. 2017; Ribeiro et al. 2017). For example, Sibiya et al. (2013) used the PPB approach in three villages in KwaZulu-Natal and reported that farmers preferred maize varieties with high yield and prolificacy, disease resistance, early maturity, white grain endosperm color, and drying and shelling qualities. A study conducted in two agro-ecological zones of Burkina Faso revealed that farmers preferred high-yielding, early-maturing, and drought-tolerant varieties (Dao et al. 2015). Using participatory on-farm trials and demonstrations, Etwire et al. (2013) reported that farmers in the Transitional and Savanna agro-ecological zones of Ghana preferred maize varieties that were early-maturing and drought-tolerant. In another study, Ribeiro et al. (2017) reported that farmers in some districts of Brong Ahafo and Ashanti Regions of Ghana preferred maize varieties that were low soil nitrogen (low-N)–tolerant and drought-tolerant as well as disease- and pest-resistant. According to earlier reports, improving farmers’ access to varieties that possess traits they prefer increases the rate of variety adoption (Heisey and Smale 1995) and diffusion through farmer-to-farmer exchange (Mulatu and Belete 2001).

**Fig. 1:**
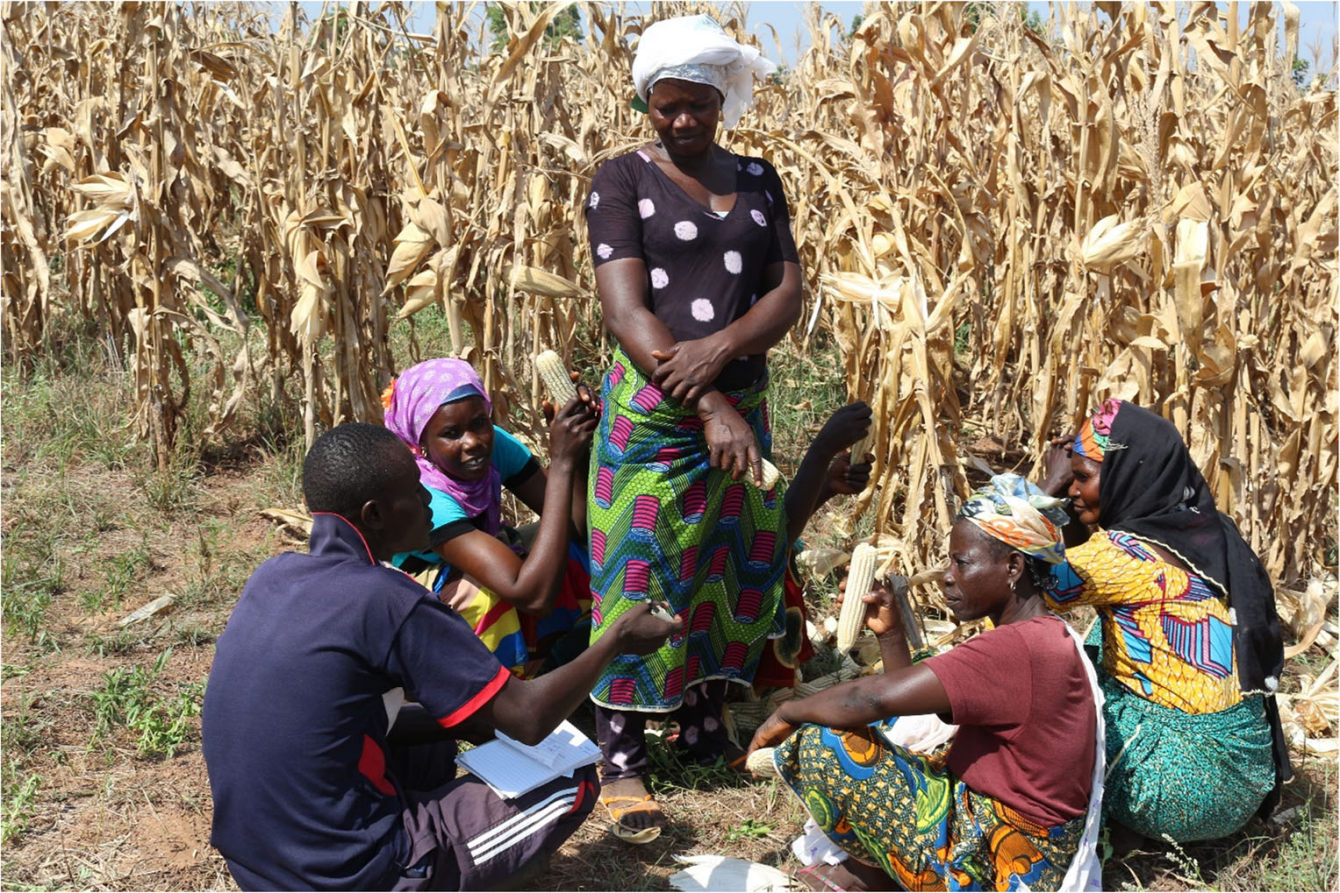
An enumerator interacting with female maize farmers during a variety selection meeting at Nyankpala in the Tolon district of Northern Region of Ghana.

Most of the earlier research on variety preferences and adoption focused mainly on the preferences of farmers without paying much attention to the needs and constraints of other value chain actors. The dynamics of food crop production in Africa has changed during the past five decades from a subsistence economy to a market economy. Moreover, the monetarization of the current economy obliges farmers to sell part of the crop to satisfy their needs (FAO 1998). This implies that farmers may produce maize to suit other end users’ needs and preferences that may be different from theirs. Therefore, the success of a variety, in terms of adoption, will depend on how well it addresses the needs of all value chain actors and not just the farmers. Analysis of the whole maize value chain by breeders will help identify critical needs in the value chain and actions needed to add more value to new varieties. The objective of this study was to identify traits preferred by farmers and other actors in the maize value chain in northern Ghana (Fig. 1). Ultimately, this information will be used to refine and guide variety development, recommendation, and deployment strategies of the Maize Improvement Programme (MIP) of the Council for Scientific and Industrial Research (CSIR)-Savanna Agricultural Research Institute (SARI).

## Materials and methods

Two experiments using the participatory rural appraisal (PRA) and participatory variety selection (PVS) methods were conducted to determine trait preferences of maize value chain actors as well as farmers’ selection criteria across the three regions of northern Ghana (Supplementary Table 1).

### First experiment: participatory rural appraisal of maize trait preferences of value chain actors in study areas

#### Sampling technique

The first experiment involved a survey of farmers, input dealers, traders, and processors along the maize value chain in northern Ghana. The farmer’s survey was conducted at the household level and targeted the major maize-producing districts of the Northern, Upper West, and Upper East Regions of Ghana. The sampling procedure adopted facilitated the generation of regionally representative samples which also covered the target areas of the MIP of CSIR-SARI. The sampling procedure combined purposive, stratified, and random techniques in three stages. At the first stage, nine project districts were purposively selected (Supplementary Table 1). Within each district, the list of maize-producing communities was generated together with the MIP of CSIR-SARI and the Ministry of Food and Agriculture (MoFA). In all, 180 farm households from 45 communities were involved in the study (Supplementary Table 1). The study also employed the use of key informant interviews targeting selected input dealers, processors, and traders, as well as focus group discussions targeting each of the three actors at the community level. In each of the 9 districts, 3 input dealers, 7 processors, and 7 traders were interviewed. A focus discussion group targeting input dealers, traders and processors was also conducted in each of the 45 selected communities.

#### Data collection

Focus group discussions were conducted to collect general information about the existing maize production system in selected communities. The data collected described the production and marketing activities of the actors, their role in the value chain, and the impact of the maize production system on livelihood and welfare outcomes. During the focus group discussions, the different value chain actors identified were asked to list the traits/characters that were considered while selecting maize varieties for their enterprises. The traits listed by the actors were grouped into four main categories as physical traits (grain color, grain size, grain yield, physiological maturity, plant height, plant aspect, cob size, cobs (ears) per plant, and cob fill); stress response traits (drought tolerance, pest and disease resistance, *Striga* resistance/tolerance and response to fertilizer application “nitrogen use efficiency”); organoleptic and grain quality traits (nutritional value, flour quantity, texture of cooked meals, consistency of cooked meals), and others (market price of grains and access to improved seeds). The actors were asked to rank the traits according to their own preferences giving reasons for their choices. The traits listed by the actors were also used to design a questionnaire, which was later used during formal interviews with farmers, traders, input dealers, and processors.

### Second experiment: participatory variety selection with farmers

#### Field experimentation

Participatory variety selection trials involving eight elite early-maturing maize hybrids were conducted in fourteen communities in Tolon, Sissala East, West Gonja, and Binduri districts in northern Ghana (Supplementary Table 1). The PVS trials started in 2014 and ended in 2016. The hybrids used in this study were developed by the International Institute of Tropical Agriculture (IITA). They possessed combined tolerance to drought and *Striga* and were early-maturing (reached physiological maturity in 90 days). The mother-baby trial approach involving researcher-managed mother trials and farmer-managed baby trials was used. Four mother trials were conducted in four farming communities across the four districts each year. The mother trials were laid out using the randomized complete block design, with each site as a replicate. In the mother trials, all the eight hybrids were planted together with two varieties nominated by farmers (farmers’ varieties 1 and 2) as local checks. In the baby trials, the eight hybrids were grouped into two sets with each set evaluated separately together with local check varieties nominated by collaborating farmers. Four baby trials were conducted in each district per year. With the help of local agricultural extension officers, lead farmers were selected from farmer-based organizations (FBOs) to manage the baby trials. The lead farmers were selected based on their experiences in maize production, access to land, and their ability and willingness to disseminate information on the target hybrids to other farmers. A plot size of 10 m × 10 m was used. Spacing between rows was 75 cm and spacing within plants was 40 cm. A compound fertilizer, NPK 15-15-15, was applied at the rate of 60 kg N ha^−1^, 60 kg P_2_O_5_ ha^−1^, and 60 kg K ha^−1^ at 2 weeks after planting (WAP). The plots were top-dressed with 30 kg N ha^−1^ at 4 WAP. Weed control was manually done at all sites when necessary to keep the plots free from weeds. All the trials were conducted under rainfed conditions.

#### Agronomic data collection

Data was recorded on the 4 inner rows of plots in both the mother and baby trials at all trial sites. Traits measured in the mother trials were plant stand, days to 50% anthesis and silking, plant and ear heights, plant and ear aspects, plant and ear numbers harvested per plot, root lodging, stalk lodging, field weight, and moisture content of grains at harvest. Data collected in the baby trials included plant vigor, plant height, ear and plant numbers harvested per plot, ear aspect, field weight, and 100-grain weight. Plant stand was the total number of plants per plot obtained soon after thinning. Days to 50% anthesis was measured as the number of days from planting to the time when 50% of plants had tassels shedding pollen. Days to 50% silking was measured as the number of days from planting to the day when 50% of plants had emerged silks. Plant height was measured as the average height of plants in centimeters (cm) from the base of the plant to the node bearing the flag leaf. Ear height was measured as the average height of the ear in centimeters (cm) from ground level to the node bearing the uppermost ear. Plants harvested per plot were measured as the total number of plants harvested per plot. Ears harvested per plot were measured as the total number of ears harvested per plot. The number of root-lodged plants was recorded as the number of plants tilted more than 30̊ angle from the ground and was scored on a scale of 1–5, where 1 = no lodging of plants; 2 =more than 20% of plants lodged; 3 = more than 40% of plants lodged; 4 = more than 60% of plants lodged; and 5 = more than 80% of plants lodged. Stalk lodging referred to the number of plants with broken stalks below the ear or the stalk bending more than 45̊ angle from the upright position. The number of stalk-lodged plants was scored on a scale of 1–5, where 1= no lodging of plants; 2=more than 20% of plants lodged; 3= more than 40% of plants lodged; 4=more than 60% of plants lodged, and 5=more than 80% of plants lodged. Field weight was measured as the weight of cobs per plot measured in kilograms. Moisture content of the grain was measured with a moisture tester (agraTronix™ MT-16) at harvest and recorded as a percentage of the grain harvested at physiological maturity. Plant aspect was scored on a scale of 1–5. Scoring was based on the assessment of overall architecture of plants in a plot as they appealed to sight, where 1 was excellent overall phenotypic appeal, 2 was very good overall phenotypic appeal, 3 was good overall phenotypic appeal, 4 was poor overall phenotypic appeal, and 5 was very poor overall phenotypic appeal. Ear aspect was scored on a scale of 1–5, where 1 was excellent with no disease/insect damage, large cobs, uniform ears, and fully filled grains, 2 was good ear with no disease/insect damage and fully filled grains, and one or two irregularities in cob size, 3 was mild insect damage, with no disease, fully filled grains, and one or two irregularities in cob size, 4 was severe disease/insect damage, with scanty grain filling, few ears, and non-uniformity of cobs, and 5 was only one or no ears. Plant vigor of all entries per plot was scored at 4, 6, and 8 WAP using a scale of 1–5, where 1 was weak plants, 2 was less vigorous plants, 3 was fairly vigorous plants, and 4 was more vigorous plants. A shelling percentage of 80% was assumed for varieties per plot. Grain yield calculated from field weight was converted to kilograms per hectare. Grain yield was adjusted to 15% moisture content. Ears per plant were obtained by dividing the total number of ears harvested per plot by the total number of plants harvested per plot.

At tasseling, 20 male and 20 female farmers from each participating community were invited to the mother trials to score for early maturity and drought tolerance. Also, the farmers were invited at harvesting, to score for cob size, grain filling, number of ears per plant, grain color, and grain yield. At each visit, the farmers were asked to rank the overall performance of the varieties based on their own indigenous criteria and preferences using a scale of 1–5, where 1 was least preferred, 2 was less preferred, 3 was fairly preferred, 4 was more preferred, and 5 was most preferred. Over 3000 farmers participated in the mother-baby trials from 2014 to 2016.

### Statistical analyses

Data on ranking of preference for maize quality attributes and traits collected from the PRA was analyzed using Kendall’s coefficient of concordance. The identified preferences were ranked from the most influential to the least influential using numerals, 1,2,3,4…… .. *n*. The mean rank score for each preferred character was computed and the factor with the least score was ranked as the least preferred quality attribute or trait while the highest score was ranked as the most preferred. The total rank score computed was then used to calculate the coefficient of concordance (*W*), which measured the degree of agreement among the respondents in the rankings. The “*W*” was estimated using the relation proposed by Tetteh et al. (2011):

1 $$ W=\frac{12\left[\sum {T}^2-\frac{{\left(\sum T\right)}^2}{n}\right]}{nm^2\left({n}^2-1\right)} $$

where *T* = the sum of the rank of factors being ranked

*m* = the number of respondents

*n* = the number of factors being ranked

*W* = the coefficient of concordance. The coefficient of concordance (*W*) was tested for significance in terms of the chi-square distribution.

Principal component analysis (PCA) was also performed for data reduction purposes. The PCA was done to identify the most important traits based on their magnitude of contributions to the total variation in the combined data sets for farmers and input dealers, and traders and processors. The PCA was performed using the relations by Anderson (1962) as follows:

Let *x* be a *p*-component random vector with mean vector *εx* = *μ* and covariance matrix *ε*(*x* − *y*)(*x* − *y*)^′^ = ∑. The variance of a linear combination *γ*^′^*x* is given by:

2 $$ \varepsilon {\left({\gamma}^{\prime }x-\varepsilon {\gamma}^{\prime }x\right)}^2=\varepsilon {\left[{\gamma}^{\prime}\left(x-\mu \right)\right]}^2=\varepsilon {\gamma}^{\prime}\left(x-\mu \right){\left(x-\mu \right)}^{\prime}\gamma ={\gamma}^{\prime}\sum \gamma $$

The linear combination normalized by *γ*^′^*γ* = 1 which has maximum variance may be called the first principal component of *x*. The linear combination uncorrelated with the first principal component and similarly normalized which has maximum variance may be called the second principal component. The other *p* − 2 principal components were similarly defined.

To obtain precise linear combinations, we used the characteristic roots and vectors of ∑.

Let *δ*_1_ ≥ … ≥ *δ*_*p*_ > 0 be the *p* characteristics roots of ∑(assumed to be positive definite). They are the roots of:

3 $$ \mid \sum -\delta I\mid =0 $$

Let *γ*_1_, …, *γ*_*p*_ are the corresponding normalized characteristic vectors; they satisfy:

4 $$ \sum {\gamma}_i={\delta}_i{\gamma}_i $$

5 $$ {\gamma}_i^{\prime }{\gamma}_i=1 $$

For the PVS trials, analyses of variance for grain yield and yield components recorded for both mother and baby trials were performed using GenStat Statistical package 16^th^ Edition (Genstat 2012). The farmers’ selection data were analyzed using simple ranking and descriptive methods in accordance with the given values.

## Results and discussion

### Socio-economic characteristics of maize value chain actors in the study areas

Maize farmers in the study areas are mainly smallholder farmers with farm sizes of 2 hectares or less. The majority of the maize-producing households (95.45%) across the study areas were headed by males (Table 1). Female participation in maize farming was generally low across the sampled population. This may have largely influenced decisions on maize productivity, since most of the decisions were often taken by household heads. This result also suggests that maize is considered as men’s crop in the study areas, and women growing maize on their own land were mostly in female-headed households. Females bear the responsibility for household tasks while males are involved in farm activities and bring cash income to the household (Doss 1999). The average number of years recorded for maize production across the sampled population was about 15 years (Table 1). This implies that continuous production of maize over the years has an influence on the knowledge of the farmers in maize production with regard to agronomic practices, variety choice, budgeting, and marketing. Maize farmers interviewed in the Northern Region had more years (16 years) of experience in production compared to farmers in the Upper East and West Regions. Experience plays a major role in the uptake of technology. Studies conducted by Martey et al. (2012) revealed that experienced household heads can take better production decisions and have greater contacts which allow trading opportunities to be discovered at lower cost. Age plays a crucial role in the adoption of improved technologies. According to Wiredu et al. (2009), younger household heads are more dynamic with regards to adoption of innovations and improved technologies for crop productivity enhancement. The average age of maize farmers in the study areas is 41 years which is lower than the national average of 50 years (PHC, 2010). This implies that farmers in the study areas can be described as relatively young and can work productively for the next two decades. The majority of the farmers (89.39%) were observed to be married (Table 1). Besides companionship, married couples in farming communities usually assist each other in farm activities. An average household size of seven was recorded across the sampled population (Table 1). On regional disaggregation, Upper West Region was observed to have the highest household size of eight. The number of people living in a household has an influence on the production output and income of the household (Al-Hassan 2008). Household members often serve as a source of family labor in the crop production system. Economically, active household members often contribute significantly towards household income.

**Table 1:** Socio-demographic characteristics of farmers and other actors in the maize value chain in northern Ghana in 2016. *NR*, Northern Region; *UER*, Upper East Region; *UWR*, Upper West Region

**Characteristics**	**NR**	**UER**	**UWR**	**Overall**	**NR**	**UER**	**UWR**	**Overall**
	**Farmers**				**Input dealers**			
Household head (%)	95.80	92.11	100.00	95.45	100.00	10.00	100.00	100.00
Years of experience in maize production	15.87	13.60	13.86	15.12	6.67	7.00	9.50	7.06.00
Sex (male, %)	99.15	97.37	100.00	98.87	100.00	100.00	100.00	100.00
Sex (female, %)	0.85	2.63	0.00	1.13	0.00	0.00	0.00	0.00
Age (years)	41.20	40.36	38.43	40.68	39.17	34.50	41.50	38.88
Married (%)	87.50	89.47	100.00	89.39	84.62	50.00	100.00	82.35
Educated (%)	37.84	13.51	9.52	29.00	84.62	50.00	50.00	82.35
Annual income (GH¢)					7069.23	4750	3800.00	6575.00
Proportion of annual income from maize trade (%)					39.90	20.00	18.42	36.09
Household size (N)	6.98	7.12	7.52	7.07				
	**Traders**				**Processors**			
Household head (%)	27.27	25.00	50.00	29.41	40.00	33.33	0.00	33.33
Years of experience in maize production	12.00	8.00	24.00	12.00	7.67	5.75	22.00	9.42
Sex (Female: %)	90.91	75.00	100.00	11.76	100.00	100.00	100.00	100.00
Age (years)	42.40	42.67	46.50	43.00	38.80	31.00	41.00	37.60
Married (%)	81.82	100.00	50.00	82.36	80.00	75.00	100.00	81.25
Educated (%)	72.73	25.00	50.00	58.83	40.00	25.00	100.00	43.75
Annual income (GH¢)	9155.45	4375.00	3200.00	7330.00	6000	1625.00	3375.00	8808.33
Proportion of annual income from maize trade (%)	84.10.00	48.57	87.50	79.29	88.06	92.31	16.29	38.29

Input dealers from the sampled population across the three regions of northern Ghana were males who were married and heads of their households. The average age of input dealers across the sampled population was 39 years (Table 1). The majority of them (82.35%) have some form of formal education. This result is consistent with those of Martey et al. (2016), who found the average age of agro-input dealers in northern Ghana to be 39 years. Similarly, a survey conducted by Krausova and Banful (2009) revealed the average age of input dealers in the Northern, Upper East, and Upper West Regions to be 38.6 years. They also reported that agro-input businesses in northern Ghana are primarily male-owned and owner-managed. Maize traders across the sampled population were predominantly females (88.24%), with a small proportion (11.76%) being males (Table 1). On regional disaggregation, majority (25%) of the male traders were found in the Upper East Region. High proportions (58.83%) of the traders were educated, having some form of formal education. These results are consistent with the findings of Badu-Apraku et al. (2003). The authors found that women dominate the maize trade in northern Ghana and that females constitute 70% of all maize sellers. The average age of maize traders across the sampled population was 43 years. This shows that maize trade is gaining grounds among the economically active age group. The majority of the traders (82%) were married, with only a smaller proportion either being single, divorced, or widowed (Table 1). Small proportion (21.49%) of the traders were household heads, which can be attributed to the large number of females engaging in the activity. The average annual income of traders across the sampled population was observed to be GH¢ 7330.00, out of which 79.29% was obtained from the sale of maize grains. Maize processors in the sampled population were generally females (Table 1). The dominance of females in maize processing could be attributed to the cultural system in the three northern regions where women are normally in charge of food preparation in the household. A small proportion of processors (33.33%) were, however, household heads. Processors had an average of nine years of maize processing experience (Table 1). The Northern Region had the highest number of processors being household heads compared to the other two regions. The majority of processors (81.25%) across the sampled population were married and have had some form of formal education. The processing activities identified in the study areas included manual or mechanical threshing, winnowing, and milling. Processors across the study areas usually processed maize into readily consumable local dishes like *Banku*, *Tuo-zaafi*, Kenkey, porridge, and *Kpaakpulo* or in raw form as floor and dough for sale.

### Trait preferences of maize value chain actors in the study areas

Kendall’s coefficient of 0.8 was obtained for farmers. This indicated that 80% of the survey respondents agreed with the ranking of traits preferred by maize farmers in the sampled population. The first most preferred trait by farmers was grain yield (Table 2). Varieties with high-yielding abilities were preferred to moderate and low-yielding ones (Supplementary Table 2). This result was highly expected, since farmers are often expectant of higher yields, and hence will prefer varieties that are high-yielding. This result corroborates earlier reports of Abdoulaye et al. (2011), Kassie et al. (2012), and Dao et al. (2015), which revealed that farmers in Ghana, Burkina Faso, Benin, Mali, Nigeria, Angola, Malawi, Zambia, and Zimbabwe consider yield potential as the most desired trait of an ideal maize variety. Grain color was considered the second most important trait in selecting maize varieties by farmers (Table 2). Varieties with white endosperm color were most preferred to varieties with yellow and mottled endosperm (Supplementary Table 2). Although both white and yellow endosperm maize varieties are grown in the study areas, it was observed that majority of the farmers grow white endosperm maize for home consumption and yellow endosperm maize for sale. These results are highly influenced by the high priority maize consumers place on grain color in Ghana. According to MoFA, white endosperm maize is the most common staple food crop produced and consumed by a vast majority of Ghanaian households. Yellow endosperm maize is primarily used in the preparation of animal feed particularly poultry feed (MoFA- SRID 2010; 2012). Empirical evidence from most surveys conducted in other African countries revealed that maize farmers and consumers will choose white endosperm maize over yellow endosperm maize (Stevens and Winter-Nelson 2008; De Groote et al. 2002; Sibiya et al. 2013). According to Ranum et al. (2014), the main reason for the preference for white endosperm maize in Africa is simply tradition; people are used to eating a white product, usually the whiter the better. Also, yellow endosperm maize is not popular in Africa because it is associated with food-aid programs and is perceived as being consumed only by poor people and animals, and some consumers perceive yellow endosperm maize to be too sweet (Ranum et al. 2014).

**Table 2:** Maize trait preferences of farmers and input dealers in northern Ghana in 2016. *NR*, Northern Region; *UER*, Upper East Region; *UWR*, Upper West Region

Character	Farmers					Input dealers				
	NR	UER	UWR	Overall	Av. Rank	NR	UER	UWR	Overall	Av. Rank
Physical traits										
Grain color	88.10	68.40	100.00	85.00	16.60	90.90	100.00	100.00	94.10	18.09
Grain size	31.10	18.40	72.70	66.10	10.28	27.30	100.00	100.00	52.90	14.50
Grain yield	87.40	63.20	95.50	83.30	16.69	90.90	100.00	100.00	94.10	14.71
Physiological maturity	79.00	78.90	100.00	81.70	14.78	90.90	100.00	100.00	94.10	18.06
Plant height	31.80	10.00	33.30	26.30	9.22					
Plant structure	4.20	2.60	0.00	3.30	5.40					
Cob size	18.20	30.00	16.70	31.60	13.28	9.10	0.00	50.00	11.80	8.79
Ears per plant	89.10	94.70	54.50	84.40	9.97	0.00	0.00	50.00	5.90	8.65
Cob fill	10.10	5.30	13.60	9.40	10.38					
Stress response traits										
Drought tolerance	21.00	2.60	0.00	15.00	12.62	18.20	75.00	50.00	35.30	12.74
Pest and disease resistance	8.40	2.60	0.00	6.70	11.77	0.00	25.00	0.00	5.90	10.03
*Striga* resistance	4.20	2.60	0.00	3.30	8.68	0.00	50.00	0.00	11.80	7.03
Nitrogen use efficiency	16.00	18.40	0.00	15.00	13.21	27.30	50.00	0.00	29.40	11.35
Organoleptic and grain quality traits										
Nutritional value	2.50	2.60	0.00	2.20	9.94					
Flour quantity	12.60	5.30	9.10	10.60	10.79	0.00	0.00	50.00	5.90	9.47
Texture of cooked meal	5.90	2.60	0.00	4.40	9.39	0.00	0.00	50.00	5.90	8.97
Consistency of cooked meal	2.50	2.60	0.00	2.20	5.56					
Other traits										
Market price of grain	12.60	7.90	81.80	20.00	7.72	45.50	75.00	25.00	100.00	13.85
Access to seed	62.20	71.10	100.00	52.80	12.85	36.40	50.00	50.00	100.00	14.62
Test statistics										
Kendall’s coefficient of concordance	0.77					0.60				
*P*-value	0.00					0.00				
Chi-square	413.02					184.80				

The period of maturity of a maize variety was ranked the third most important trait by the farmers interviewed (Table 2). Early-maturing varieties were the most preferred by a large proportion (79.21%) of farmers compared to late and intermediate maturing varieties (Supplementary Table 2). This result could have been influenced by the geological location of the respondents. Northern Ghana experiences a uni-modal tropical monsoon rainfall pattern, allowing one growing season with an average annual rainfall of 900 to 1100 mm (MoFA – SRID 2012). Rainfall amount and distribution have often been very erratic in northern Ghana due largely to the impacts of global climatic change. Given that maize production in northern Ghana is predominantly carried out under rainfed conditions, the use of early-maturing varieties will increase farmers’ resilience to climate variability. Dao et al. (2015) reported that maize farmers in Burkina Faso generally grow early-maturing varieties in the northern zone of the country where the rainy season is relatively shorter but grow late-maturing varieties in the southern zone where the rainy season is of longer duration. Unlike medium to late-maturing varieties, early-maturing varieties can escape terminal drought and heat stress. They often mature before the onset of severe terminal drought and several modern early-maturing varieties also possess drought tolerance genes making them most suitable for drought-prone areas in northern Ghana and other countries in sub-Saharan Africa (Bänziger et al. 2000; Badu-Apraku and Fakorede 2017). The preference for early-maturing varieties by farmers in the study areas corroborates earlier findings that revealed that early-maturing varieties are increasingly becoming important in drought-prone areas of West Africa (Benin, Ghana, Mali, and Nigeria) (Abdoulaye et al. 2011) and also in Southern Africa (Angola, Malawi, Mozambique, Zambia, and Zimbabwe) (Kassie et al. 2012) due to increasing unreliability and unpredictability of weather patterns. Cob size was ranked the fourth most important trait ahead of other agronomic traits like plant height, number of cobs per plant, and cob fill (Table 2). The majority of the farmers (82.61%) were of the view that varieties with bigger cobs produced higher yields compared to cobs of medium and small sizes (Supplementary Table 2). Farmers consider cob size as a key yield-related trait, and they usually use cob size for visual selection of varieties with high yield. Findings of De Groote et al. (2002), Kasoma et al. (2020), and Yatai et al. (2020) revealed that top varieties that receive farmers’ highest score for grain yield are usually the same as those that receive the highest ranks for cob size. Nitrogen use efficiency was ranked as the fifth most important trait by farmers ahead of other stress response traits like drought tolerance (ranked 7th), pest and disease resistance (ranked 8th), and *Striga* resistance (ranked 16th) (Table 2). Varieties with high nitrogen use efficiency were preferred to moderate- and low-N-efficient ones (Supplementary Table 2). Northern Ghana has poor soils with declining soil fertility (Abdoulaye et al. 2011; Etwire et al. 2013). Ellis-Jones et al. (2012) identified low and declining soil fertility and high cost of agro-inputs as major crop production constraints in northern Ghana. Farmers’ preference for nitrogen use efficient varieties could be a risk coping strategy, a perceived economical way of improving their maize yields without overly depending on chemical fertilizers. The findings of Ribeiro et al. (2017) revealed low soil fertility as the most dominant constraint in maize productivity in Wenchi and Ejura Sekyedumase districts in Ghana. In Angola, Mozambique, and Zimbabwe, a variety’s performance under poor soil fertility is an important trait considered along with yield potential in the selection of an ideal maize variety by farmers (Kassie et al. 2012).

In the study areas, the most preferred traits by input dealers, in order of importance, were grain color, days to maturity, grain yield, access to seed, and grain size. Input dealers preferred white endosperm varieties, early-maturing varieties, and high-yielding varieties with grain of large size. Kendell’s coefficient of concordance indicated that 60% of the sampled input dealers agreed with each other on the ranking of the traits (Table 2). The trait attributes preferred by input dealers were similar to the preferences of farmers. Grain yield, grain color, and days to maturity were the top three traits considered by both farmers and input dealers in selecting maize varieties across the three regions in northern Ghana. Although preference ranks by both actors were different, the attributes of these traits preferred by both actors were the same (Supplementary Table 2). This result is expected as input dealers are not direct users of the maize varieties and thus will only commercialize varieties with traits frequently demanded by their main customers, who are the farmers. Access to seeds was ranked the fourth and sixth most important determinant of variety adoption by input dealers and farmers, respectively. At the community level, farmers complained about the unavailability of high-quality seeds of high-yielding and stress-tolerant varieties in their communities to enhance crop production. The report of Ellis-Jones et al. (2012) identified lack of improved seeds as the first and second most important constraints to crop production in Upper West and Northern Regions, respectively. A report by USAID-EAT (2012) indicated that less than 10% of maize farmers in the Northern Region have access to improved seeds. The results of the present study suggest that farmer’s poor access to improved seed is a major constraint to maize production and adoption of new varieties in rural communities in northern Ghana.

**Fig. 2:**
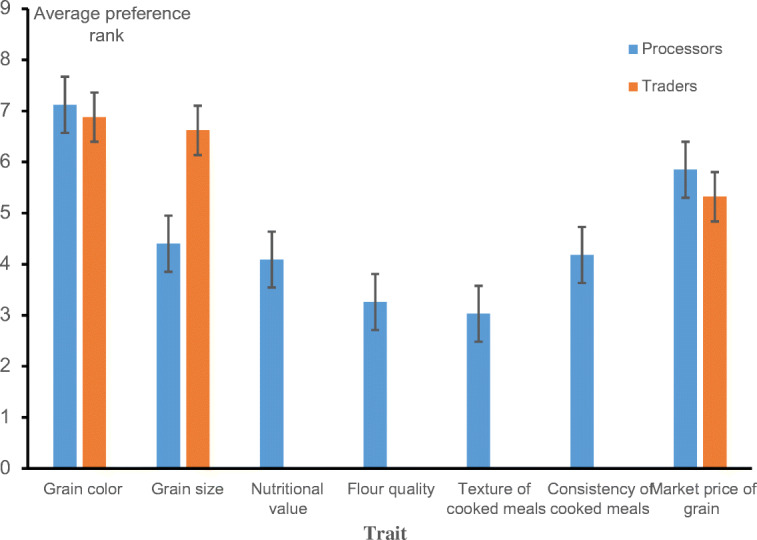
Traits considered by traders and processors in Northern Ghana when selecting maize varieties and their rank averages. Trader Kendal’s statistics: (*W* = 0.464, *P* = 0.000, Chisq. = 55.220); processors Kendal’s statistics: (*W* = 0.369, *P* = 0.000, Chisq. = 49.943).

About 50% of traders and 40% of processors in the sampled population agreed with the ranking of the traits they prefer, respectively (Fig. 2). For traders, grain color, grain size, and market price of grains were ranked as the most important traits, in that order (Fig. 2). Processors also ranked grain color as the first most important trait they consider in selecting maize varieties for processing (Fig. 2). Grain size, market price of grain, consistency of the cooked meals, and their nutritional value were considered as the second to fifth most important traits, respectively. Traders and processors highly preferred maize varieties with white endosperm to those that have yellow and mottle endosperm (Supplementary Table 2). A lot of attention was paid to grain color because the traders were of the view that consumers consider it most when purchasing maize grains. According to the processors, consumers usually find maize product of white endosperm maize more appealing compared to those prepared with yellow endosperm maize. Preference for grain size was high among majority of the input dealers, traders, and processors (Table 3 and Fig. 2). According to traders and input dealers, larger grains/seeds usually attract high market price compared to medium and small grains/seeds. According to processors, maize varieties that have larger grains produce more flour compared to varieties that have smaller grain size. However, some of the respondents were of the view that medium-sized grains have less chaff and high flour quantity when milled compared to large-sized grains. The trait preferences of processors were influenced by local product preferences and processing methods. The most important organoleptic traits considered by processors were consistency of the cooked meals (4th), the flour quantity (5th), and texture of the cooked meals (6th) (Fig. 2). Processors preferred maize grains that give heavy starch when processed. The majority of the processors in the sampled population preferred maize meals with smooth texture over those that are rough, hard, lumpy, and gritty. They also preferred their cooked meals not to change in consistency after cooking (Supplementary Table 2). These organoleptic traits are linked to grain qualities of a variety (i.e., texture of the endosperm, size and shape, and thickness of the pericarp) and its behavior in processing (hulling or milling) (FAO 1998). These results imply that processors in northern Ghana prefer mostly maize varieties with dent grain type and to some extent semi-dent grain types. This is because dent grains have a higher percentage of soft starch which can easily be milled to fine flour, while flint maize has a hard and vitreous endosperm and a gritty texture (Sylvie De Buck 2017). Empirical evidence of variety adoption patterns in Africa indicates that farmers prefer local varieties that give better results after processing than improved varieties with high grain yield potential but give poor processing results: In Togo, maize varieties that have soft grains are more appreciated since they give fine flour with minimum grinding. Improved varieties such as NH1F, Mexico 8049, or TZSR having similar processing qualities are well adopted by farmers in Togo. On the other hand, a variety like La Posta, needing 2 or 3 passes through the mill, is more costly to process and therefore is of little interest (FAO 1998). In Benin, consumers prefer to make “lifin” (a traditional maize flour) using local varieties that have more friable grains which give fine flour with lower damaged starch content when milled while the improved varieties with vitreous and hard grains give coarse flour when processed (Nago et al. 1997).

**Table 3:** Correlation matrix of important principal components of traits considered by farmers and input dealers, and traders, and processors in Northern Ghana when selecting maize varieties.

Trait	Farmers and input dealers								Traders and processors			
	Component								Component			
	1	2	3	4	5	6	7	8	1	2	3	4
Grain color	−0.10	−0.02	−0.41	0.40	−0.31	0.21	−0.22	−0.37	−0.61	0.25	−0.08	0.61
Grain size	0.25	−0.02	0.21	0.25	0.23	−0.02	−0.65	0.06	−0.75	−0.32	−0.12	−0.04
Grain shape	−0.03	−0.02	0.11	0.05	0.13	0.81	0.12	−0.18	−0.05	0.65	0.31	−0.02
Nutritional value	0.17	0.26	−0.29	0.56	0.11	−0.17	−0.30	0.01	−0.20	0.22	0.68	−0.42
Flour quantity	0.49	0.29	0.38	−0.20	0.07	0.00	−0.10	−0.06	0.19	−0.63	0.23	0.32
Texture of cooked meal	−0.21	0.38	−0.01	0.49	0.25	−0.17	0.34	0.26	0.36	0.20	−0.69	−0.34
Consistency of cooked meal	0.54	0.33	0.10	0.02	0.33	−0.17	0.17	−0.23	0.53	0.42	0.02	0.54
Market price of grain	−0.18	−0.07	0.70	−0.06	0.04	0.19	−0.01	−0.06	0.63	−0.30	0.32	0.05
Plant height	0.44	−0.16	0.06	0.10	0.14	0.28	−0.15	0.51				
Plant structure	−0.34	0.15	0.43	0.26	0.29	−0.08	0.15	0.17				
Cob size	0.17	−0.65	−0.09	0.25	0.32	0.02	0.32	0.16				
Ears per plant	0.35	−0.44	0.06	0.10	0.37	−0.08	0.24	−0.33				
Cob fill	0.34	−0.49	−0.30	−0.16	0.03	0.21	−0.02	0.15				
Physiological maturity	0.09	−0.31	0.51	0.32	−0.40	−0.12	0.10	−0.22				
Drought resistance	0.65	0.29	0.14	0.11	−0.18	0.14	−0.08	0.28				
Pest and disease resistance	−0.05	0.49	−0.38	−0.01	0.29	0.43	0.15	−0.04				
*Striga* resistance	0.63	0.29	0.02	−0.49	0.11	−0.10	0.03	−0.23				
Grain yield	0.31	−0.06	−0.12	−0.18	−0.51	−0.07	0.28	0.36				
Nitrogen use efficiency	0.44	0.43	−0.24	0.23	−0.20	−0.05	0.30	−0.10				
Access to seed	−0.11	0.42	0.36	0.21	−0.34	0.27	0.14	0.04				

Market price of grains was identified as an important criterion used by input dealers, processors, and traders in the study areas in the selection of maize varieties. It had the highest rankings among processors (second important trait), while traders and input dealers ranked it as the third and sixth important trait, respectively (Tables 1 and 2, and Fig. 2). Fluctuation in market prices was preferred to constant market prices by all three actors. At the community level, market prices of grains are often set by traders with little to no participation by other actors. Prices are determined mainly based on the transport cost and time of the year. Both farm-gate and wholesale prices increase as distance to major markets and transport costs increases. Also, prices are low soon after harvesting and higher during the off-season (Sarpong and Anim-Somuah 2015). Traits like grain size and grain color also influence market prices and must be considered carefully in variety development. Yellow endosperm maize is usually sold at a higher price than white endosperm maize.

### Principal component analysis of traits considered by maize value chain actors in selecting maize varieties

The PCA of trait preferences of both farmers and input dealers revealed 8 principal components (PCs) having eigenvalue greater than 1 (Supplementary Table 3). The 8 PCs explained 64.23% of the total variation within the combined dataset on traits preferred by farmers and input dealers in selecting an ideal maize variety (Supplementary Table 3). Eleven out of the 21 traits studied were identified as the main traits that influenced the selection criteria of farmers and input dealers: In order of importance, these traits were drought tolerance, *Striga* resistance, cob size, market price of grain, days to maturity, grain color, nutritional value, grain yield, grain shape, grain size, and plant height (Table 3). This result suggested that although grain yield is among the most preferred traits of both farmers and input dealers, traits like drought tolerance, *Striga* resistance, cob size, market price of grain, days to maturity, grain color, and nutritional value were of high priority to both actors than grain yield during variety selection. Trait preferences tacitly indicate the objectives and priorities of actors, and preferences are also dictated by the opportunities and constraints value chain actors face in their enterprise selection and management (Kassie et al. 2012), Therefore, farmers’ strong interest in traits such as drought tolerance, *Striga* resistance, and early maturity, and also in market price of grains, grain color, and nutritional value implies that farmers are not only interested in high-yielding varieties but are also most interested in varieties that are adapted to their production constraints and also suitable to the preferences of local consumers. Yield loss due to drought stress is estimated at 49–70% of the annual maize produced in SSA. The incidence and severity of drought are predicted to rise due to global climate change. The over-reliance of farmers on rainfall increases the vulnerability of maize production systems to climate variability and change (Kogo et al. 2020; Ndlovu et al. 2020). Therefore, drought-tolerant varieties will play an important role in adaptation to climate change (Kogo et al. 2020; Ndlovu et al. 2020). Farmers and input dealers’ strong preference for drought-tolerant varieties reflects their perceived vulnerability to drought and also shows their awareness of the potential benefits of drought-tolerant varieties. Community analyses of cereal-based farming systems in Northern, Upper West, and Upper East Regions of Ghana by Ellis-Jones et al. (2012) identified drought and *Striga* infestation as the fourth and sixth most important constraints to crop production in Upper West and Northern Regions, respectively. In a community survey by Abdoulaye et al. (2011), drought was frequently cited by farmers as a major constraint to maize production in Benin, Ghana, and Nigeria. In Malawi, Zambia, and Zimbabwe, drought is reported as the most important challenge on the livelihoods of maize farmers, whereas in Angola and Mozambique, drought is reported as the second most important challenge to farmers, next to sickness and mortality of a family member. In all the countries above, strategies adopted by farmers to cope with drought include planting of early-maturing and drought-tolerant varieties.

The PCA of traits preferences of both traders and processors revealed 4 principal components (PCs) having eigenvalues greater than 1 (Supplementary Table 3). The 4 PCs explained 68.04% of the total variation within the combined dataset on traits considered important by traders and processors in selecting an ideal maize variety (Supplementary Table 3). Grain size, market price of grain, grain color, and consistency of cooked meal had the highest loading on PC1, respectively (Table 3). Thus, they are the traits with the highest influence on the selection criteria of traders and input dealers. Traits such as grain shape and flour quantity and nutritional value and texture of cooked meals were identified, respectively, as the second and third orders of important traits by both actors (Table 3). The PCA results revealed that farmers, input dealers, traders, and processors in the sampled population consider grain color, grain size, market price of grains, nutritional value, and consistency of cooked meals when selecting a maize variety for their enterprises (Table 3). As discussed earlier, preferences for all of these five traits by the different value chain actors are dictated by local consumers’ preferences. This result corroborated the findings of FAO-AGSE (1994), Heisey and Smale (1995), and Smale et al. (2011) that indicated that although the importance of a trait has different implications depending on the role of an actor in a value chain, as long as a typical value chain revolves around the needs of consumers, consumer preferences will always determine the overall acceptance of a variety. The overall implication of the results of the PCA analyses is that consumer preferences are important but need to be adjusted with production constraints.

#### Mean grain yield and other agronomic performance of the early-maturing hybrids evaluated in the participatory variety selection trials

The mean grain yield of the varieties across mother and baby trials was 2.8 t/ha. The top three yielding hybrids across the four districts were EYH-29, EYH-42, and EYH-19, respectively. EYH-29, EYH-42, and EYH-19 out-yielded the best local check (Farmer variety 2) by 45.3%, 34%, and 20.8%, respectively (Table 4). EYH-29, EYH-42, and EWH-19 produced the highest yield in West Gonja and Binduri districts. In Tolon and Sissala East districts, EYH-29, EYH-42, and EWH-29 were the top three yielding hybrids. Higher grain yield was associated with taller plants, more ears per plant, and good plant and ear aspects (Table 4).

**Table 4:** Mean agronomic performance of early-maturing hybrids evaluated on-farm in West Gonja (WG), Binduri (BI), Tolon (TL), and Sissala East districts in 2014 to 2016. NB: *, ** = significant at 5 and 1% level of probability, respectively; ns, not significant; EWH, early white hybrid; EYH, early yellow hybrid; EQWH, early quality protein maize hybrid

Hybrid	Grain yield (t/ha)				Plant height (cm)				Ears per plant				Ear aspect			
	WG	BI	TL	SE	WG	BI	TL	SE	WG	BI	TL	SE	WG	BI	TL	SE
EQWH-18	2.9	2.5	2.9	3.0	167.0	99.0	144.9	143.8	1.0	0.8	1.0	1.0	1.3	2.0	1.0	1.5
EWH-3	3.0	1.7	2.9	3.0	159.3	98.0	129.3	135.3	1.0	0.9	0.9	1.0	2.0	2.3	1.0	1.9
Farmer variety 1	2.1	1.6	1.8	1.5	180.7	112.0	204.0	173.8	0.6	0.7	0.9	0.6	4.0	3.7	2.0	4.1
EYH-42	3.8	2.9	3.5	4.0	189.0	133.7	162.2	169.7	1.0	0.9	1.0	1.0	2.0	2.7	1.3	2.1
EYH-29	3.9	3.7	3.9	3.9	174.7	140.0	161.3	166.6	1.0	0.9	1.1	1.0	1.0	1.0	1.3	1.8
EYH-1	2.8	2.5	2.8	2.9	156.0	132.7	137.9	149.3	1.0	1.0	1.0	1.0	1.7	2.7	1.0	1.9
Farmer variety 2	2.5	2.6	2.7	2.8	192.0	156.3	158.6	177.4	1.0	0.7	0.8	0.9	3.0	4.0	4.0	4.0
EYH-19	3.0	3.3	3.4	3.1	163.3	156.3	143.5	162.1	1.0	1.0	1.0	1.0	2.0	2.7	1.3	2.1
EWH-28	1.8	1.9	2.4	2.0	117.0	140.0	131.8	136.1	0.9	0.9	1.0	1.0	1.7	2.0	1.3	1.8
EWH-29	3.2	2.4	3.5	3.4	165.7	138.7	157.8	161.8	1.0	0.9	1.0	1.0	1.7	2.0	1.0	1.4
Mean	2.9	2.5	3.0	3.0	166.47	144.8	145.9	157.3	1.0	0.9	1.0	1.0	2.0	2.7	1.7	2.2
LSD (5%)	0.4*	0.3*	0.4*	0.2*	11.5*	15.0**	20.0*	15.0**	0.1**	0.1*	0.1*	0.1**	1.0**	1.1**	1.0^ns^	2.0*

#### Varietal traits and genotypes preferred by producers

**Fig. 3:**
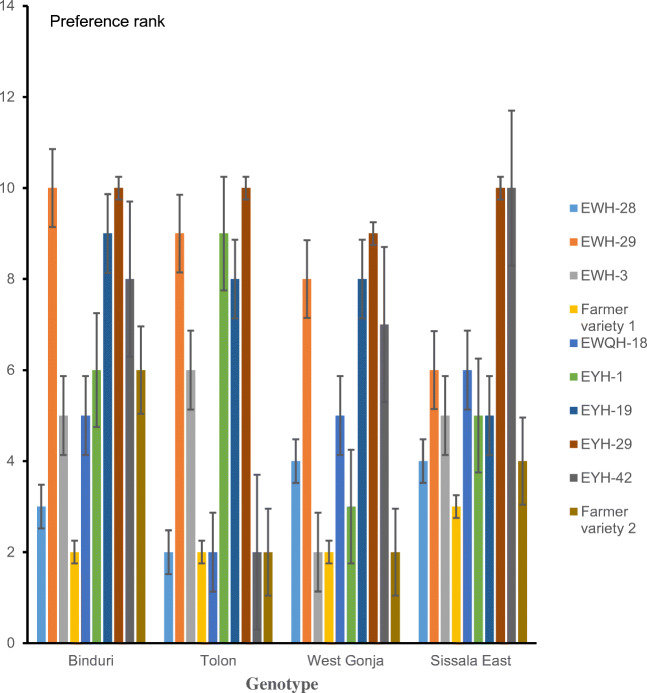
Overall preference ranks of eight early-maturing hybrids and farmers’ varieties averaged across gender and four districts in Northern Ghana between 2014 and 2016.

**Fig. 4:**
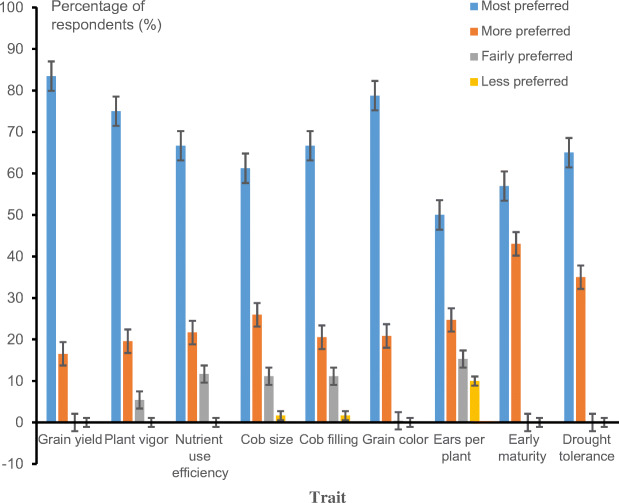
Importance of the different traits farmers consider when selecting maize varieties in Northern Ghana.

Farmers’ preferences for the eight hybrids varied across districts (Fig. 3). In Sissala East district, farmers ranked EYH-29 and EYH-42 as the most preferred hybrids. EYH-29 was the most preferred hybrid by farmers in West Gonja and Tolon districts. In Binduri district, farmers ranked both EWH-29 and EYH-29 as the most preferred hybrids. Across districts, the most preferred hybrids had higher grain yield, early maturity, bigger and fully filled cobs with multiple ears per plant, and excellent ear aspect (Table 4). Varieties with vigorous growth and thick green leaves at the vegetative stage were also preferred by farmers. Farmers considered varieties with thick green leaves at the vegetative stage to have a better response to nitrogen fertilizer use. The most common selection criteria used by farmers across districts and gender included grain yield (83.5%), grain color (78.8%), plant vigor (75%), nutrient use efficient (66.7%), cob filling (66.7%), drought tolerance (65%), and cob size (61.3%) (Fig. 4). The results of both the PVS trials and PRA survey identified grain yield, grain color, nutrient use efficiency, cob filling, drought tolerance, and cob size as very important traits in the variety selection criteria of farmers (Table 2 and Fig. 4). Therefore, they must be considered as key traits in maize breeding programs. Most importantly, the preferred attributes of the individual traits by farmers as identified in this study must be taken into consideration during variety development to enhance the adoption of new improved varieties by farmers in the study areas. Generally, the conduct of the PVS trials afforded the research team of the MIP of CSIR-SARI an opportunity to interact with participating farmers and witness at first-hand different selection criteria applied/used by maize farmers in the study areas. The PVS trials helped to identify hybrids which were considered superior to some released varieties adopted by farmers in Tolon, West Gonja, Binduri, and Sissala East districts. In 2017, the hybrids EYH-29 and EWH-29 were recommended by the MIP of CSIR-SARI to the National Variety Release and Registration Committee for release to stakeholders in Ghana. All two hybrids were officially released and registered in the national catalog of crop varieties released in Ghana in 2019. The release names of EYH-29 and EWH-29 are CSIR-Denbea and CSIR-Similenu, respectively. Other information on these two hybrids are available in Adu et al. (2017).

## Conclusions and implications

The value chain actors identified in the study areas were primarily farmers, input dealers, traders, and processors. The commonest and most important traits considered by all four actors when selecting maize varieties were grain color, grain size, and market price of grains, nutritional value, and consistency of cooked meals. According to the four actors, these are the traits consumers look for in a maize variety, and therefore, they control the marketing of maize grains and its products in their communities. In addition to the above traits, farmers, and input dealers also considered drought tolerance, *Striga* resistance, cob size, days to maturity, and grain yield as important traits when selecting an ideal maize variety. The PVS trials also identified plant vigor, nutrient use efficiency, and cob filling as important traits to farmers. Most national/public maize breeding programs in West and Central Africa including the program in CSIR-SARI already consider drought tolerance, *Striga* resistance, early maturity, and grain yield as high priority traits (Badu-Apraku and Fakorede 2017; Badu-Apraku et al. 2012). However, for our breeding programs to be more responsive to the needs of all value chain actors, the other key traits identified in this study and their respective preferred attributes should be targeted as well. These traits should be incorporated into new varieties being developed, to make them attractive to all actors along the maize value chain. This will contribute significantly to increased adoption of improved maize varieties among smallholder farmers across the three regions of northern Ghana. While new varieties are being developed, old varieties could be replaced by the more recently released and registered early-maturing drought- and *Striga*-tolerant hybrids such as EYH-29 (CSIR-Denbea) and EWH-29 (CSIR-Similenu) that are more suited to the preferences of actors in northern Ghana.

## Supplementary information

ESM 1 (DOCX 22.7 kb)

## Data Availability

The datasets generated during the present study are available from the lead and corresponding authors on request.
