# Supplementary material for: Trait profile of maize varieties preferred by farmers and value chain actors in northern Ghana
Source: Agron Sustain Dev. 2021 Jul 6;41(4):50. doi: 10.1007/s13593-021-00708-w (PMC8550044; doi:10.1007/s13593-021-00708-w)
Supplement: Supplementary file 1 — (DOCX 22.7 kb) [file 13593_2021_708_MOESM1_ESM.docx]

**Supplementary Table 1:** **Names of regions, districts and communities where the study was conducted in 2016**

| **Region** | **District** | **Community** |
| --- | --- | --- |
| Northern | Gushegu | Pulo, Sampebga, YisheiKpugi, Gumonaayili and Kpatili |
| Northern | Cheriponi | Jakpa, Gballo, Nyangbandi, Nawieku, Ando- Nyamanu, Tachiku, Sangbana, Ando and Kanani |
| Northern | Sawla-Tuna-Kalba | Gindabour, Poliyiri, Kpongeri No.2, Kalba, Gorkon, Jacheriyiri, Jentilpe and Nyangakura |
| Northern | Tolon | Nyankpala, Kpalsogu and Tingoli |
| Northern | Mion | Salankpang, Dijow and Sang |
| Northern | West Gonja | Damongo |
| Upper East | Garu-Tempane | Kpalsagu, Akarateshi, Konkomada, Nissbuliga, Bantafarigu and Kpatua |
| Upper East | Binduri | Nayoko No. 2, Kpaligu and Gumyoko |
| Upper West | Sissala East | Chinchang, Dimajan, Kupulma, Katinia, Silbelle and Kong |

**Supplementary Table 2:** **Preference rank averages of attributes of traits considered by farmers and other value chain actors in the study areas when selecting maize varieties**

| **Attributes of trait** | **Farmers** | **Input dealers** | **Attributes of trait** | **Farmers** | **Input dealers** | **Traders** | **Processors** |
| --- | --- | --- | --- | --- | --- | --- | --- |
| Grain yield |  |  | Grain colour |  |  |  |  |
| High | 72.6 | 94.1 | White | 86.6 | 94.1 | 94.1 | 88.24 |
| Moderate | 7.7 | 0.0 | Yellow | 18.5 | 11.8 | 52.9 | 47.06 |
| Low | 3.0 | 0.0 | Mottled | 0.0 | 0.0 | 0.0 | 0.0 |
| Physiological maturity | | | Grain size |  |  |  |  |
| Early | 80.1 | 94.1 | Large | 38.2 | 52.9 | 52.9 | 40.0 |
| Late | 2.0 | 6.3 | Medium | 3.0 | 31.3 | 29.4 | 14.29 |
| Intermediate | 7.7 | 12.5 | Small | 0.0 | 0.0 | 0.0 | 0.0 |
| Plant height |  |  | Market price of grain | | | | |
| Tall | 38.1 | 11.8 | Constant | 1.8 | 0.0 | 0.0 | 0.0 |
| Short | 3.3 | 0.0 | Fluctuating | 30.4 | 56.3 | 82.4 | 56.3 |
| Medium | 5.2 | 6.3 | No trend | 8.6 | 0.0 | 0.0 | 0.0 |
| Cob size |  |  | Flour quantity | |  |  |  |
| Big | 43.6 | 41.2 | Heavy | 9.5 | 0.0 |  | 14.3 |
| Small | 0.6 | 0.0 | Light | 1.8 | 100.0 |  | 0.0 |
| Medium | 0.0 | 0.0 | Intermediate | 3.0 | 100.0 |  | 14.3 |
| Pest and disease resistance | | | Consistency of cooked meal | | | | |
| Highly resistant | 4.5 | 5.9 | Hardens | 0.0 | 0.0 |  | 0.0 |
| Moderately resistant | 0.6 | 0.0 | No change | 0.6 | 0.0 |  | 7.1 |
| Susceptible | 0.0 | 0.0 | Heavy | 0.6 | 0.0 |  | 0.0 |
| Drought tolerance | | | Nutritional value | | | | |
| Highly tolerant | 9.6 | 18.8 | Quality protein maize | 1.1 | 0.0 |  | 0.0 |
| Moderately tolerant | 4.7 | 0.0 | Normal maize | 0.6 | 0.0 |  | 0.0 |
| Susceptible | 1.2 | 0.0 |  |  |  |  |  |
| Nitrogen-use efficiency | | | Texture of cooked meal | | | | |
| High | 15.7 | 41.2 | Rough | 0.6 | 0.0 |  | 7.1 |
| Moderate | 0.6 | 6.3 | Smooth | 9.5 | 6.3 |  | 40.0 |
| Low | 0.0 | 0.0 | Hard | 0.6 | 0.0 |  | 0.0 |
| Access to improved seeds | | | Soft | 0.0 | 6.3 |  | 0.0 |
| High | 51.7 | 52.9 | Lumpy | 2.4 | 0.0 |  | 0.0 |
| Low | 1.2 | 0.0 | Gritty | 0.0 | 0.0 |  | 0.0 |
| Moderate | 1.2 | 0.0 |  |  |  |  |  |

**Supplementary Table 3:** **Eigenvalues and total variance explained by principal components of traits considered by farmers and input dealers, and traders and processors in northern Ghana when selecting maize varieties**

| **Farmers and input dealers** | | | |
| --- | --- | --- | --- |
| **Component** | **Initial Eigenvalues** | | |
|  | **Total** | **% of Variance** | **Cumulative %** |
| 1 | 2.72 | 12.93 | 12.93 |
| 2 | 2.29 | 10.92 | 23.85 |
| 3 | 1.87 | 8.90 | 32.74 |
| 4 | 1.66 | 7.89 | 40.63 |
| 5 | 1.44 | 6.85 | 47.48 |
| 6 | 1.27 | 6.05 | 53.53 |
| 7 | 1.17 | 5.57 | 59.10 |
| 8 | 1.08 | 5.14 | 64.23 |
| **Traders and processors** | | | |
| **Component** | **Initial Eigenvalues** | | |
|  | **Total** | **% of Variance** | **Cumulative %** |
| 1 | 1.82 | 22.77 | 22.77 |
| 2 | 1.35 | 16.89 | 39.66 |
| 3 | 1.21 | 15.16 | 54.82 |
| 4 | 1.06 | 13.22 | 68.04 |
